# Performance of a UV-A LED system for degradation of aflatoxins B_1_ and M_1_ in pure water_:_ kinetics and cytotoxicity study

**DOI:** 10.1038/s41598-020-70370-x

**Published:** 2020-08-10

**Authors:** Judy Stanley, Ankit Patras, Brahmaiah Pendyala, Matthew J. Vergne, Rishipal R. Bansode

**Affiliations:** 1 Food Biosciences and Technology Program, Department of Agricultural and Environmental Sciences, College of Agriculture, Tennessee State University, Nashville, TN 37209 USA; 2 Department of Pharmaceutical Sciences, Department of Chemistry and Biochemistry, Lipscomb University, Nashville, TN 37204 USA; 3 Center for Excellence in Post-Harvest Technologies, North Carolina Research Campus, North Carolina Agricultural and Technical State University, Kannapolis, 28081 NC USA

**Keywords:** Biochemistry, Microbiology, Environmental sciences, Diseases, Engineering, Optics and photonics

## Abstract

The efficacy of a UV-A light emitting diode system (LED) to reduce the concentrations of aflatoxin B_1_, aflatoxin M_1_ (AFB_1_, AFM_1_) in pure water was studied. This work investigates and reveals the kinetics and main mechanism(s) responsible for the destruction of aflatoxins in pure water and assesses the cytotoxicity in liver hepatocellular cells. Irradiation experiments were conducted using an LED system operating at 365 nm (monochromatic wave-length). Known concentrations of aflatoxins were spiked in water and irradiated at UV-A doses ranging from 0 to 1,200 mJ/cm^2^. The concentration of AFB_1_ and AFM_1_ was determined by HPLC with fluorescence detection. LC–MS/MS product ion scans were used to identify and semi-quantify degraded products of AFB_1_ and AFM_1_. It was observed that UV-A irradiation significantly reduced aflatoxins in pure water. In comparison to control, at dose of 1,200 mJ/cm^2^ UV-A irradiation reduced AFB_1_ and AFM_1_ concentrations by 70 ± 0.27 and 84 ± 1.95%, respectively. We hypothesize that the formation of reactive species initiated by UV-A light may have caused photolysis of AFB_1_ and AFM_1_ molecules in water. In cell culture studies, our results demonstrated that the increase of UV-A dosage decreased the aflatoxins-induced cytotoxicity in HepG2 cells, and no significant aflatoxin-induced cytotoxicity was observed at UV-A dose of 1,200 mJ/cm^2^. Further results from this study will be used to compare aflatoxins detoxification kinetics and mechanisms involved in liquid foods such as milk and vegetable oils.

## Introduction

Filamentous fungi invading various feed crops produces toxic secondary metabolites called mycotoxins which possess a serious threat to consumer health^1^. Aflatoxins are highly cytotoxic and carcinogenic secondary metabolites, produced predominantly by *Aspergillus flavus* and *Aspergillus paraciticus*, especially in the tropical and subtropical regions as hot and humid climatic conditions are optimal for mold growth and toxin production^2,3^. Aflatoxins are difuranocoumarin derivatives formed from the polyketide pathway and include a group of 17 aflatoxins, among which AFB_1_ is the predominant and is highly toxic^4^. Aflatoxin contamination in animal feed results in their carry-over in foods of animal origin such as eggs and milk. The major AFB_1_ metabolite in milk, AFM_1,_ is formed due to the action of hepatic cytochrome P450-dependent polysubstrate monoxygenase enzyme superfamily as a result of the hydroxylation of the fourth carbon of the terminal furan ring. In dairy cows, the biotransformation rate of AFB_1_ to AFM_1_ ranges from 0.3 to 6.2%^5,6^.

Aflatoxins are detrimental upon ingestion, inhalation, and skin contact; and the consequences of aflatoxin infection are collectively called aflatoxicosis. The biotransformation of AFB_1_ in liver results in the production of AFB_1_-8,9-epoxide which is highly related to the incidence of hepatocellular carcinoma^6,7^_._ The carcinogenicity of AFM_1_ is approximately one-tenth of that of AFB_1_ The International Agency for Research on Cancer in 1993 classified AFB_1_ under Group 1 carcinogen and AFM_1_ under group 2B carcinogen^8^; therefore, some international organizations have enacted stringent regulations to mitigate aflatoxin contamination in food and feed. The maximum residue limit (MRL) of aflatoxins for human consumption ranges from 4 to 30 µg/kg^9^. The European Union has the strictest standards, the AFB_1_ level should not exceed 2 µg/kg in edible oils and AFM_1_ levels should not exceed 0.05 µg/kg^9^. The United States Food and Drug Administration specified maximum acceptable limits of 20 µg/kg for total aflatoxins and 0.5 µg/kg for AFM_1_ in human food and milk^9,10^.

Aflatoxin contamination in food and feed can be reduced by various processing methods. For example, aflatoxin contamination during growth and storage of grains can be reduced by employing various pre- and post-harvest technologies. Several aflatoxin decontamination methods include destruction by physical methods including heating at high temperatures; selective separation using adsorbents such as reduced graphene-oxide-gold nanoparticles^11^; chemical modification using several acids, bases, and oxidizing agents; and biological decontamination using enzymes and fermentation^1^. Despite their efficacy, each method presents certain challenges such as utilization of chemicals, adverse impacts on the nutritional and sensorial attributes of the food, and difficulties in scale-up, thereby limiting their use in food industry^12^. Exploration of safe, cost-effective, and novel food processing technologies with an objective of achieving maximum inactivation of aflatoxins with minimal effect on the quality of food helps to address these challenges^13^. Examples of alternative innovative food processing technologies include electromagnetic irradiation, advanced-packaging materials, and dielectric heating^1^. Pulsed light technology, which effectively degrade aflatoxins, utilizes broad spectra of white light, that includes ultra-violet, visible and infra-red^14^. Studies show that the light intensity and the UV spectrum greatly influence the degradation of aflatoxins more than the visible and infra-red spectrum^15^.

UV irradiation has been demonstrated in literature as an effective physical method to inactivate chemical contaminants, and micro-organisms through photolysis and DNA damage, respectively^16–20^. This non-thermal technology is efficient in degrading aflatoxins because of their photosensitivity^21^. Dominant sources of UV treatment such as low pressure and medium pressure mercury lamps were used to degrade aflatoxins. In a study, 98% reduction of AFB_1_ in water was observed at an UV dose of 4,880 mJ/cm^2^ using a medium pressure UV lamp which emits irradiation between 200 and 360 nm wavelength^16^. Similarly, 100% reduction of AFB_1_ in peanut oil was observed when UV intensity (220–400 nm) of 800 mJ/cm^2^ was used for 30 min^22^. In a separate study, irradiation at 365 nm showed nearly 100% degradation of AFM_1_ in milk after 60 min of exposure to 100 W lamp^23^. As an alternative to mercury containing traditional UV lamps, alternative sources of UV light such as UV-LEDs and excimer lamps are being studied for their application in food industry as they have several advantages like mercury free, high energy efficiency, constant light intensity and prolonged lifetime^24,25^.

The presence of mycotoxins in food and feed has been investigated extensively. But few studies reported the occurrence of mycotoxins in surface, ground and wastewaters due to contamination from agricultural fields^26,27^. Paterson et al., in 1997 first detected the presence of aflatoxins in cold storage water tank^28^. Aflatoxin B_2_ was the most often detected mycotoxin present in bottled water followed by aflatoxin B1, aflatoxin G1 and ochratoxin A^29^. Even though, the levels of aflatoxins found in water is low (ng/L), long term exposure may cause health risk. Until now, no reports are available on UV degradation of aflatoxins in pure water. AFB_1_ and AFM_1_ have absorption maxima at 362 nm which elevate their susceptibility for degradation when exposed to light around 362 nm^30^. Hence, we hypothesize irradiation of aflatoxins using UV-A light at 365 nm could be efficient to degrade aflatoxins in water via photo irradiation. The key points related to application of UVA light in aflatoxin reduction and detoxification include the analysis of kinetics, quantum yield and understanding the cytotoxic behavior of AFB_1_ and AFM_1_ degradation products for liver cells. Furthermore, a major issue in many UV studies is that they do not account the absorbance of the test fluid^22,23^; hence, the present study rectifies this problem by accounting for fluid optics, and corrections for UV fluence gradients.

In this study, a custom-built laboratory scale batch reactor using an UV-A LED source which emits at a peak wavelength of 365 nm was used. This study is carried out to determine the degradation kinetics and the possible degradation mechanism of AFB_1_ and AFM_1_ in a pure water, without the hindrance of other biomolecules which may result in UV-A attenuation. This study also assesses the cytotoxicity of UV-A treated samples which contain residual AFB_1_ and AFM_1_ and their degradation products in water using human hepatoma cell line (HepG2).

## Material and methods

### Chemicals and reagents

AFB_1_ and AFM_1_ were procured from LKT laboratories, Inc (St. Paul MN, USA). Human hepatoma cells (HepG2; ATCC HB-8065), Eagel’s minimum Essential Medium (EMEM; ATCC 30-2003), and fetal bovine serum (FBS; ATCC 30-2020) was purchased from American Type Culture Collection (ATCC, Manassas, VA).

### Preparation of Aflatoxins standards in ultrapure water

Aflatoxin standard solutions were prepared by dissolving AFB_1_ and AFM_1_ in methanol. Working solutions of AFB_1_ and AFM_1_ with initial concentrations of 1 µg/mL and 2 µg/mL, respectively were prepared in ultrapure water before being exposed to UV-A irradiation. As methanol is found to be toxic to cells, for cytotoxicity studies, AFB_1_ and AFM_1_ were dissolved in DMSO, each with initial concentration of 25 µg/mL.

### Light emitting diodes irradiation system

A UV-A LED (IRTRONIX, Torrence, CA, USA) which emits at a peak wavelength of 365 nm, mounted on top of a quasi-collimated bench scale reactor, was used to perform the irradiation experiments. Five mL of the test solution was dispensed in a 10 mL beaker and placed above the magnetic stirrer. The test solution was continuously stirred to ensure uniform dose distribution and cold water (4 °C) was continuously circulated to prevent increase in temperature during irradiation at higher doses. Central irradiance incident on the surface of the test solution was measured with the help of a high sensitivity spectrometer (QE Pro series, Ocean Optics, Dunedin, FL, USA). Average irradiance was calculated by taking into account the absorption of the test solution at 365 nm. The absorbance was determined using a double beam Cary100 Spectrophotometer connected to a 6-inch single integrating sphere (Agilent Technology, Santa Clara, CA, USA). The volume-averaged irradiance was evaluated by incorporating corrections factors (reflection factor, petri factor, divergence factor, water factor) as per the standard method described by Bolton and Linden^31^. Average irradiance was divided by target UV-A dose to obtain specific exposure times. Total UV-A doses of 0, 300, 600, 900 and 1,200 mJ/cm^2^ were delivered to AFB_1_ and AFM_1_ test solutions and each treatment was done in triplicates in a randomized order.

1 $$Average\;fluence\left( {{\text{mW/cm}}^{2} } \right) = Incident\;fluence \times \left( {\frac{{1 - 10^{{ - \left( {{\text{a}} \times {\text{d}}} \right)}} }}{{\ln \left( {10} \right) \times {\text{a}} \times {\text{d}}}}} \right) \times \left( {\frac{{\text{L}}}{{{\text{L}} + {\text{D}}}}} \right)$$

2 $$UV - A\;Dose\,\left( {{\text{mJ/cm}}^{2} } \right) = Average\;fluence \times Treatment\;time\,\left( {\text{s}} \right)$$

where ‘*Incident fluence*’ is the incident irradiance at the surface of the liquid, ‘a’ is the absorption coefficient per cm at 365 nm, d is the depth of fluid in the beaker, and ‘L’ is the distance from center of lamp source to the lower meniscus of the surface of the liquid.

### HPLC analysis of AFB_1_ and AFM_1_

Separation and quantification of irradiated AFB_1_, AFM_1_ and their degradation products were carried out using a HPLC System (Shimadzu Scientific Instruments, Columbia, MD, USA) following the method described by Patras et al.^16^ with slight modification. A reversed-phase C_18_ column (Phenomenex, CA, USA) with configuration 150 mm × 4.6 mm 2.6 µm, maintained at 37 °C was used as stationary phase. Separation was achieved with the mobile phase consisting of water/acetonitrile/methanol in the ratio of 120:75:30, under isocratic flow mode with a flow rate of 1 mL/min. Aflatoxins were detected using a Shimadzu RF-20A fluorescence detector with the excitation and emission wavelengths set at 365 and 450 nm respectively. The calibrated concentration for the HPLC method validation of AFB_1_ and AFM_1_ ranged between 0.25 and 1.0 µg/mL, 0.5 and 2.0 µg/mL for AFB_1_ and AFM_1_, respectively.

### Aflatoxins degradation analysis LC–MS/MS

The identification of Aflatoxin B_1,_ M_1_ and the respective degraded products was carried out with an LCMS method using a Shimadzu Prominence XR UHPLC system connected to a Shimadzu LCMS 8040 triple-stage quadrupole mass spectrometer with a chromatographic and mass spectrometric method described previously^16^. The column used for separation was a Phenomenex Kinetex 2.6 C18 column (50 × 2.1 mm, 2.6 µm). The injection volume was 5 µL. Control and experimental samples were injected, and data acquired in the scan mode to search for degraded products. Once degraded products were identified, corresponding selected ion monitoring (SIM) MS methods were developed for monitoring the parent and degraded product ions: AFB_1_ (*m*/*z* 313), AFB_1_ degraded products (*m*/*z* 303 and *m*/*z* 331), AFM_1_ (*m*/*z* 329), and AFM_1_ degraded products (*m*/*z* 347). The dwell time was 10 ms for all SIM events. Control and experimental samples were injected and analyzed. The chromatographic peak areas were determined and compared to controls with Shimadzu LabSolutions V5.89 software (Shimadzu, Columbia, MD, USA).

### Cell cytotoxicity analysis

Cell cytotoxicity analysis was carried out using the method described by Patras et al.^16^ with slight modification. The HepG2 cells (American Type Culture Collection (ATCC); HB-8065) with a cell plating density of 2 × 10^5^ cells per well were seeded in a 12-well plate containing 10% (v/v) fetal bovine serum (FBS) in Eagle’s minimal essential medium (EMEM). After 24 h, the cells were washed thrice with PBS and serum starved overnight in EMEM containing 1% FBS. Following serum starvation, the cells containing 1 mL media were exposed to 10% (v/v) of untreated and treated AFB_1_ and AFM_1_ test solutions at a final concentration of 2.5 µg/mL. After 48 h, the cells were measured for viability using XTT assay (ATCC, Manassas, VA) as per the manufacturer’s protocol.

### Kinetic modelling and data analysis

Log-linear reduction model available in the GInaFiT tool (a freeware add-in for Microsoft Excel)^32^ was used to describe the UV-A degradation kinetics of aflatoxins (B_1_ and M_1_). This model provides good fit to data in which the inactivation exhibits first order kinetics and goodness of fit parameters including R^2^, root mean square error, and rate constants were evaluated. The model is given in the following equation,

3 $$C = C_{0} e^{{ - k_{max} \times D}}$$

where ‘C’ is the initial concentration of aflatoxin, ‘C_0_’ is the concentration of aflatoxin at dose D, ‘k’ is the degradation rate constant and D is the UV-A dose delivered.

For identification purposes, the expression was reformulated as,

4 $$log_{10 } \left( C \right) = log_{10 } \left( {C_{0} } \right) - \frac{{k_{max} \times D}}{{ln_{10} }}$$

A balanced design with three replicates for each treatment was exposed to the selected UV-A treatment. Each sample was independent and assigned randomly to a treatment. One-way ANOVA with Tukey's HSD multiple comparison tests were performed to assess the effects of UV-A in SAS statistical computing environment (SAS, 2016). Data are presented as means ± standard deviation from the mean. Statistical significance was tested at 5 percent significance level.

## Results and discussion

In this study, a novel UV-A LED which emits with the peak wavelength of 365 nm was used. The UV-A LED system employed in this study emits peak irradiation at 365 nm. Table 1 shows the characteristics (optical properties) of the test solutions prior to UV-A irradiation. The absorbance of AFB_1_ and AFM_1_ were 0.094 and 0.101/cm and ultraviolet transmittance (%) was calculated as 80.5 and 79.3/cm.

**Table 1:** Optical properties and treatment parameters of AFB_1_ and AFM_1_ in ultrapure water under UV-A radiation.

Parameters	Aflatoxin B_1_	Aflatoxin M_1_
Irradiance (mW/cm^2^)	10.26	10.26
Absorbance (Au/cm)	0.094	0.101
Transmittance (%T/cm)	80.5	79.250
Exposure time (s)	39, 78, 117, 156	39, 79, 118, 157
Delivered dose (mJ/cm^2^)	300, 600, 900, 1,200	300, 600, 900, 1,200

**Figure 1:**
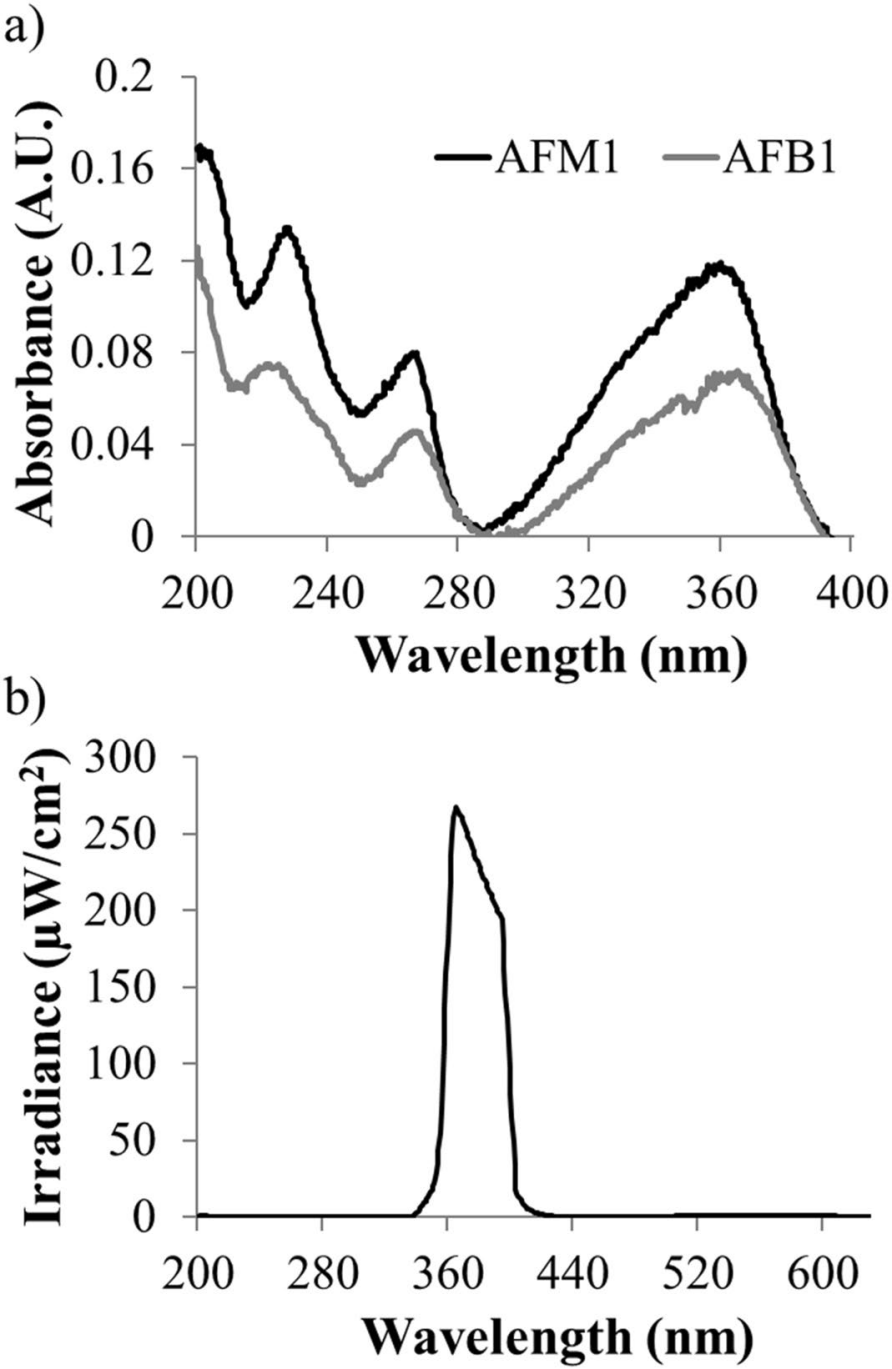
(**a**) Measured absorption spectra of AFB_1_ and AFM_1_ in ultrapure water using Cary100 spectrophotometer (**b**) Measured spectral irradiance of UV-A LED using Ocean optics QE Pro spectrometer equipped with UV–visible optical fiber.

The absorption spectrum of AFB_1_ and AFM_1_ observed in the ultraviolet region of the electromagnetic spectrum and the relative emission spectra of the lamp are shown in Fig. 1. The data show AFB_1_ and AFM_1_ strongly absorbs UV-A irradiation at 362 nm (Fig. 1a). The peak emission of the UV-A light source was noticed at 365 nm (Fig. 1b).

### UV-A degradation kinetics of AFB_1_ and AFM_1_

**Figure 2:**
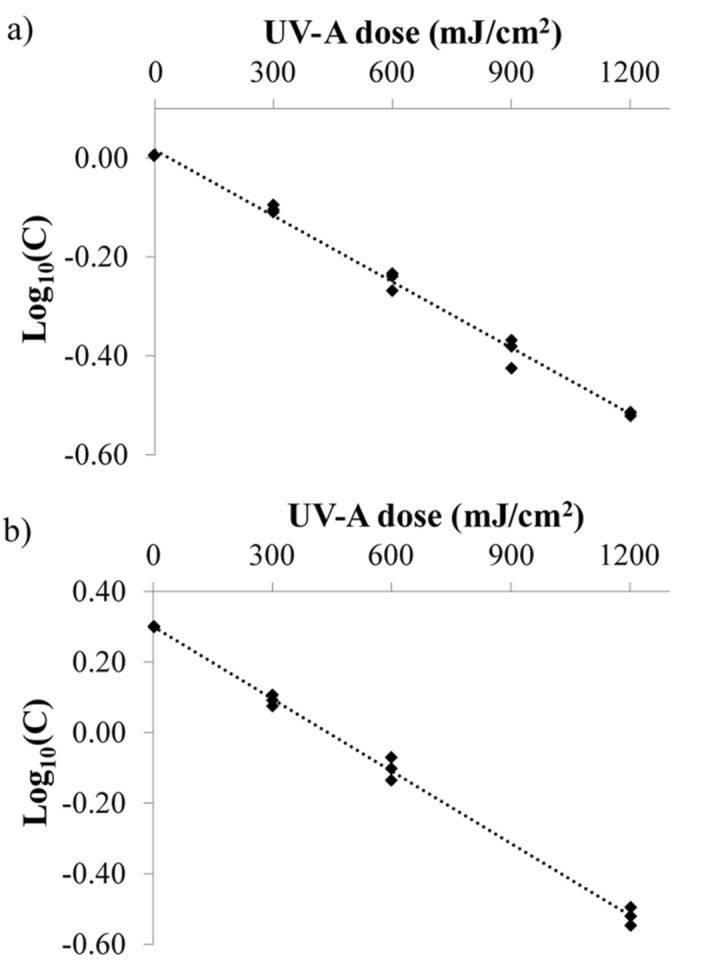
Degradation kinetics of (**a**) AFB1 (**b**) AFM_1_ in ultrapure water at different UV-A dose levels.

In this study, Aflatoxins solutions AFB_1_ and AFM_1_ in ultrapure water were irradiated at different UV-A doses ranging from 0 to 1,200 mJ/cm^2^. UV-A degradation of AFB_1_ (C_0_ = 0.997 ppm) and AFM_1_ (C_0_ = 2 ppm) in ultrapure water are compared in Fig. 2.

The degradation reaction followed first-order kinetics for AFB_1_ and AFM_1_ and both were reduced by more than 70%. At UV-A doses of 300, 600, 900 and 1,200 mJ/cm^2^ AFB_1_ reduced by 22.0 ± 1.32, 43.9 ± 2.34, 59.6 ± 2.66 and 70.0 ± 0.27 percent respectively. Similarly, a reduction of 36.0 ± 2.70, 57.5 ± 4.10, 76.3 ± 0.90, 84.0 ± 1.95 percent of AFM_1_ was observed at UV-A doses 300, 600, 900 and 1,200 mJ/cm^2^ respectively. Log_10_(C) is plotted against the UV-A doses delivered. Table 2 shows the kinetic parameters of the irradiation experiment. Log linear trend was observed which indicates that the degradation of AFB_1_ and AFM_1_ in ultrapure water under UV-A irradiation follows first-order kinetics (R^2^ > 0.99), given by the Eq. (4). The kinetic rate constant for AFB_1_ degradation in water was 0.001 cm^2^/mJ, which is ~ 1.6 times lesser than the kinetic constants for AFM_1_ (0.0016 cm^2^/mJ).

**Table 2:** Kinetic parameters of AFB_1_ and AFM_1_ under UV-A irradiation.

Parameters	Aflatoxin B_1_	Aflatoxin M_1_
R^2^	0.99	0.99
K_max_ (cm^2^/mJ)	0.001	0.0016
Half-life (mJ/cm^2^)	693	433
Quantum yield	0.0043	0.0057

Quantum yield is a fundamental photochemical parameter, which explains the photochemical fate of the compound, under controlled conditions where light absorption and change in target compound concentration are properly quantified^33,34^. Quantum yield is the number of defined events occurring per photon absorbed by the system^35^. For a photochemical reaction, quantum yield ɸ(λ) at a wavelength λ is given by,

5 $$\upphi \left(\uplambda \right) = \frac{moles\;of\;reactant\;consumed\;or\;products\;formed}{{Einsteins\;absorbed}}$$

Einsteins absorbed during the UV-A treatment is easily obtained by the following equation;

The energy of a single photon is quantified by the following law:

6 $$E = \frac{h c}{\lambda }$$

where h = Planck constant (6.63 × 10^−34^ J s), c = light speed (3 × 10^8^ m/s), λ = wavelength (m). In our case

7 $$E = \frac{{6.63 \times 10^{34} \left[ {{\text{Js}}} \right] \times 3 \times 10^{8} \left[ {\frac{{\text{m}}}{{\text{s}}}} \right]}}{{3.65 \times 10^{ - 7} \,{\text{m}}}} = 5.44222E - 19J$$

Absorbed energy is given by:

8 $$Einsteins\;absorbed \left( {{\text{E}}\,{\text{m}}^{3} } \right) = \frac{{(Absorbed\;energy/Photon\;energy\;at\;365\,{\text{nm}})}}{energy\;in\;1\;mole\;of\;photons}$$

9 $$Absorbed\;energy \left( {{\text{J}}\,{\text{m}}^{3} } \right) = \left( {\frac{{Power\;absorbed \times treatment\;time \left( {\text{s}} \right)}}{{Volume\,\left( {{\text{m}}^{3} } \right) }}} \right)$$

Power absorbed is obtained by following equation:

10 $$Power\;absorbed\,\left( {\text{W/s}} \right) = \left( {I_{0} } \right) \times \left( A \right) - I_{0} \left( A \right)10^{{ - \left( {A_{365} \times d} \right)}}$$

where $I_{0}$ is surface irradiance (W/m^2^); $A$ is the area (m^2^), $A_{365}$ is the absorbance value at 365 nm (base10/m), $d$ is the fluid depth (m).

The quantum yields of AFB_1_ and AFM_1_ in ultrapure water were determined based on Table 3.

**Table 3:** Quantum yield calculation of AFB_1_ and AFM_1_ in ultrapure water under UV-A radiation.

UV-A dose (mJ/cm^2^)	Irradiance (W/m^2^)	Treatment time (s)	Absorbed energy (J/m^3^)	Einsteins absorbed (E/m^3^)	Aflatoxin (mol/m^3^)	Quantum yield
**AFB**_**1**_						
300	102.6	39	79,236	0.24	0.0025	0.0105
600	102.6	78	158,473	0.48	0.0018	0.0038
900	102.6	117	237,709	0.73	0.0013	0.0018
1,200	102.6	156	316,945	0.97	0.0010	0.0010
					Average	0.0043
**AFM**_**1**_						
300	102.6	39	84,171	0.26	0.0032	0.0039
600	102.6	79	170,500	0.52	0.0022	0.0026
900	102.6	118	254,671	0.78	0.0014	0.0014
1,200	102.6	157	338,842	1.03	0.0010	0.0010
					Average	0.0057

The average quantum yields of AFB_1_ and AFM_1_ in ultrapure water were found to be 4.25 × 10^−3^ and 5.74 × 10^−3^ respectively. The quantum yield of AFM_1_ in ultrapure water is 1.35 times more than that of the quantum yield of AFM_1_. Comparing the UV-A photolysis rate constants, and quantum yield, it is found that AFM_1_ is more susceptible to UV-A photolysis than AFB_1_.

Under UV-A radiation, AFB_1_ acts as a photosensitizer and results in the formation of reactive oxygen species through the involvement of triplet excited state, by either Type I or Type II mechanism. In Type I mechanism, electron transfer occurs from triplet aflatoxin to molecular oxygen resulting in the formation of superoxide anion radical. In Type II mechanism, energy is transferred from triplet aflatoxin to molecular oxygen leading to singlet oxygen formation. These reactive species react with the aflatoxins and form oxidized products^36^. Various authors have studied the degradation kinetics of aflatoxins. For example, Patras et al*.* observed 98% reduction of AFB_1_ with a UV dose of 4,880 mJ/cm^2^ in ultrapure water when irradiated using medium pressure lamp which emits irradiation between 220 and 400 nm^16^. Similarly, Liu et al*.* used UV lamp emitting at 220–400 nm with an irradiance of 800µw/cm^2^ and observed thorough reduction of AFB_1_ in peanut oil after 30 min of UV exposure, the degradation followed first order kinetics^22^. Mao et al*.* used a UV-A lamp (lamp power = 100 W; irradiance = 55–60 mW cm^2^) which emits at 365 nm to treat AFB_1_ in peanut oil, the authors observed ≈96% reduction after 30 min of UV-A exposure (UV dose equivalent = 108,000 mJ/cm^2^)^37^. Diao et al*.* tested a continuous flow UV-A reactor (lamp power = 36 W; irradiance = 6.4 mW/cm^2^; flow rate = 0.55 L/min) to treat AFB_1_ in peanut oil at 365 nm and observed 88.74% reduction after 40 min of UV-A exposure^38^. It should be noted that the authors did not report the UV-A doses; a common discrepancy in many studies. From the above studies, it is quite evident that optical based techniques (i.e. irradiation) can degrade the aflatoxins efficiently due to their photosensitivity.

### AFB_1_ and AFM_1_ degradation products

**Figure 3:**
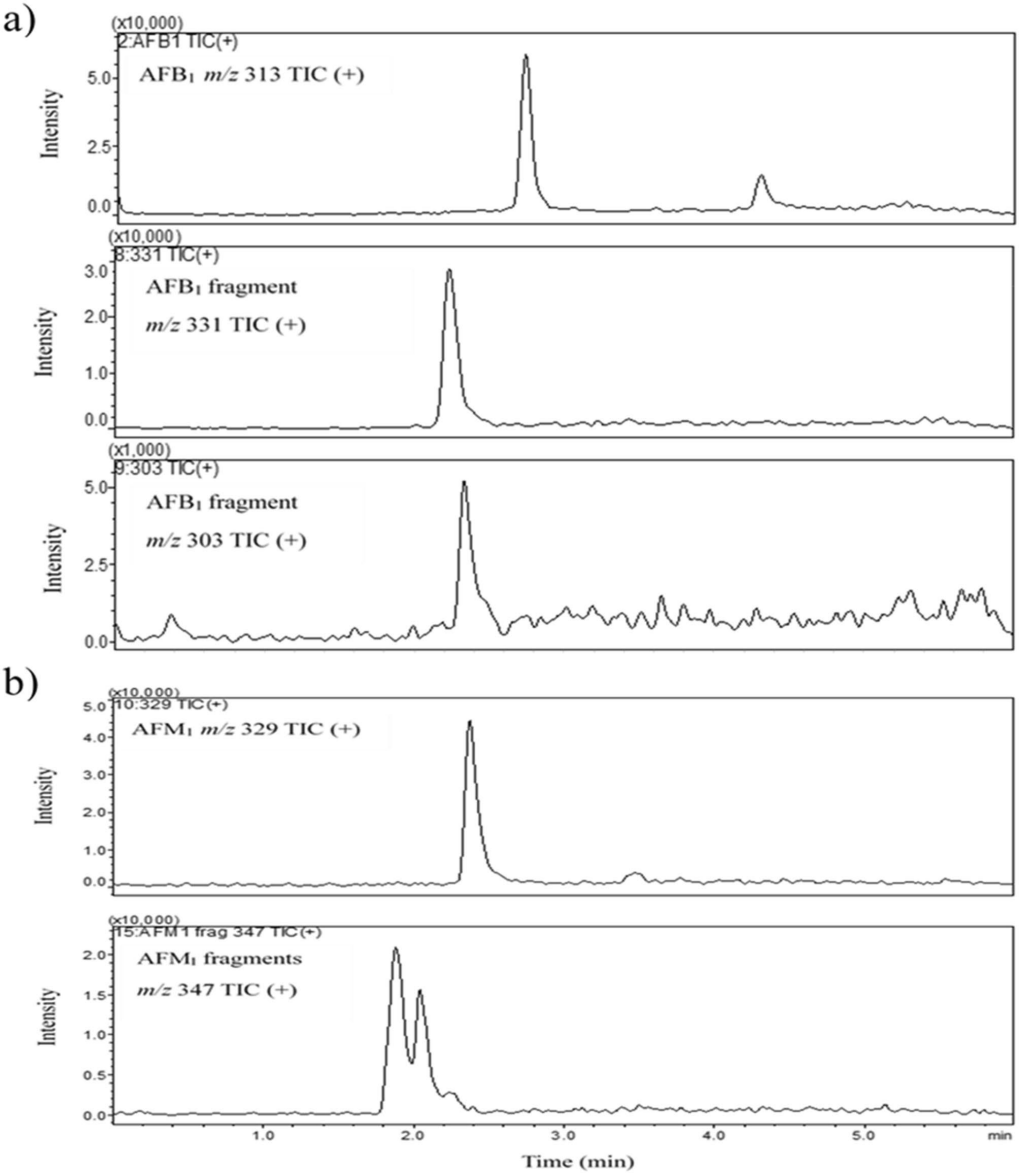
LCMS single ion monitoring (SIM) total ion chromatograms (TIC) of (**a**) AFB_1_ and (**b**) AFM_1_ samples treated at UV-A dose 1,200 mJ/cm^2^.

LC–MS scans were used to search for degraded products of AFB_1_ and AFM_1_ in water after UV-A irradiation, and are given in Fig. 3.

LC–MS single ion monitoring (SIM) chromatographic peaks were analyzed as a qualitative approach to approximate the amount of degraded products produced by determining the areas of (SIM) chromatographic peaks. Since authentic standards of the degraded products are not readily available, a quantitative approach is not possible. When compared to controls, LC–MS peak areas for AFB_1_ and AFM_1_ decreased and the peak areas of the degraded products increased significantly with increase in the UV-A dose. Representative SIM chromatograms and the LC–MS peak areas for AFB_1_ and AFM_1_ are presented in the supplemental information (Figure S1).

**Figure 4:**
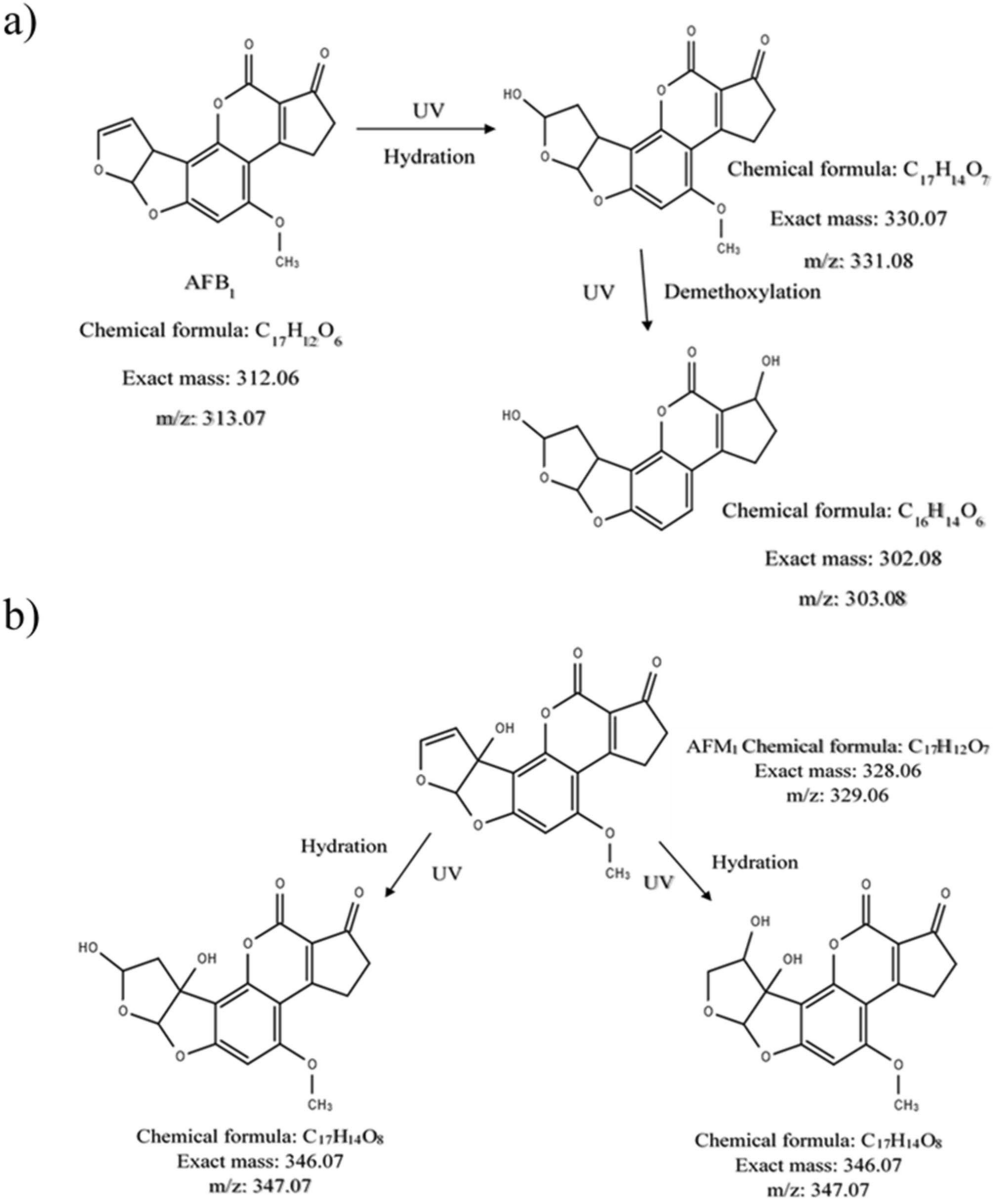
Proposed UV-A light degradation mechanism of (**a**) AFB_1_ and (**b**) AFM_1_ in ultrapure water.

The structure of the degraded products of AFB_1_ and AFM_1_ was identified and the possible degradation pathway is proposed based on them, as given by Fig. 4. Two degradation products, P_1_ and P_2_ were observed for AFB_1_ in water. Hydration and demethoxylation were the main fragmentation pathways of AFB_1_. The double bond equivalence (DBE) of AFB_1_ is 12. The photolysis product C_17_H_14_O_7_ (P_1_, *m*/*z* 331.08) was the result of hydration on the double bond of terminal furan ring of AFB_1_, with DBE of 11 which is one less than AFB_1_. The structure of product C_17_H_14_O_7_ (P_1_, *m*/*z* 331.08) is similar to that of Aflatoxin B_2A,_ whose toxic potential is comparatively less than AFB_1,_ and is inactive with respect to toxicity to ducklings and is non-lethal to chick embryos^39,40^. Product C_16_H_14_O_6_ (P_2_) (m/z 303) was the result of hydration on furan ring and demethylation on the side chain of benzene. The DBE of P_2_ is 10, which is 2 less than AFB_1_. The AFB_1_ degraded products had retention times of 2.2 and 2.3 min.

Two degraded products with similar chemical formula C_17_H_14_O_8_ (m/z 347.07) were observed for AFM_1_ and were found to be structural isomers of each other. Hydration was observed to be the main fragmentation pathway which occurred at the double bond of terminal furan ring. The DBE of both the products were found to be 11, which is one less than AFM_1_ (DBE of AFM_1_ = 12). The AFM_1_ degraded products had retention times of 1.8 and 2.0 min. The DBE of all the photolysis products of both AFB_1_ and AFM_1_ were found to be less than that of their parent molecules, implying that double bond addition reactions occurred.

The UV-A irradiation results in the formation of reactive species, which react with AFB_1_ and AFM_1_ resulting in photolysis. Hence, the structure of all photolysis products formed due to the reaction of free radicals with AFB_1_ and AFM_1_ is almost similar to that of their parent molecules^41^. The double bond in the terminal furan ring and the lactone ring in the coumarin moiety are considered to be the most toxicological sites of AFB_1_^42^_._ While AFB_1_ is not mutagenic, it is bioactivated by undergoing epoxidation of double bond in the furan ring results in the formation of AFB_1_-8,9-epoxide and it is the key active site for its toxic and carcinogenic activities as the aflatoxin-DNA and the aflatoxin-protein interactions occur^6,7^. From the proposed structure of the degradation products, it is shown that the double bond in the terminal furan ring is removed in the degradation products of both AFB_1_ and AFM_1_. Hence, based on quantitative structure–activity relationships, it is evident that the toxicity of the photolysis products is reduced compared to the toxicity of AFB_1_ and AFM_1_.

### Cytotoxicity analysis

Aflatoxins are reported to reduce cell survival in various cultured cells, especially HepG2 cells as liver is the target organ of aflatoxins^43,44^_._ Several literature studies have demonstrated aflatoxins‐induced oxidative stress damage, and its effect in hepatotoxicity^45–47^. AFB_1_ can induce reactive oxygen species (ROS) to cause oxidative stress, also cause genetic alterations prone to DNA damage and alter mitochondrial permeability^46,48,49^. Liu et al*.* (2016) reported precise mechanism of AFB1 induced on hepatotoxicity in primary broiler hepatocytes, AFB_1_ impaired mitochondrial functions by inducing reactive oxygen species and oxidative stress resulting in the activation of caspase-3 and caspase-9-induced apoptosis through mitochondrial signal pathway in addition to maintaining proper redox balance^50^. On the other hand, AFM_1_ is a detoxification product of AFB_1_, and AFM_1_ showed only 10% of mutagenicity when compared to AFB_1_^51^. The metabolic fate of AFM1 resulted to be similar to that of AFB1, with the difference that AFM1 represents a poorer substrate for epoxidation, thus explaining the differences in genotoxicity potencies. Moreover, it has been reported that Cytochrome P450 (CYP) activation is not required to AFM1 to exert cytotoxic effects^52^.

**Figure 5:**
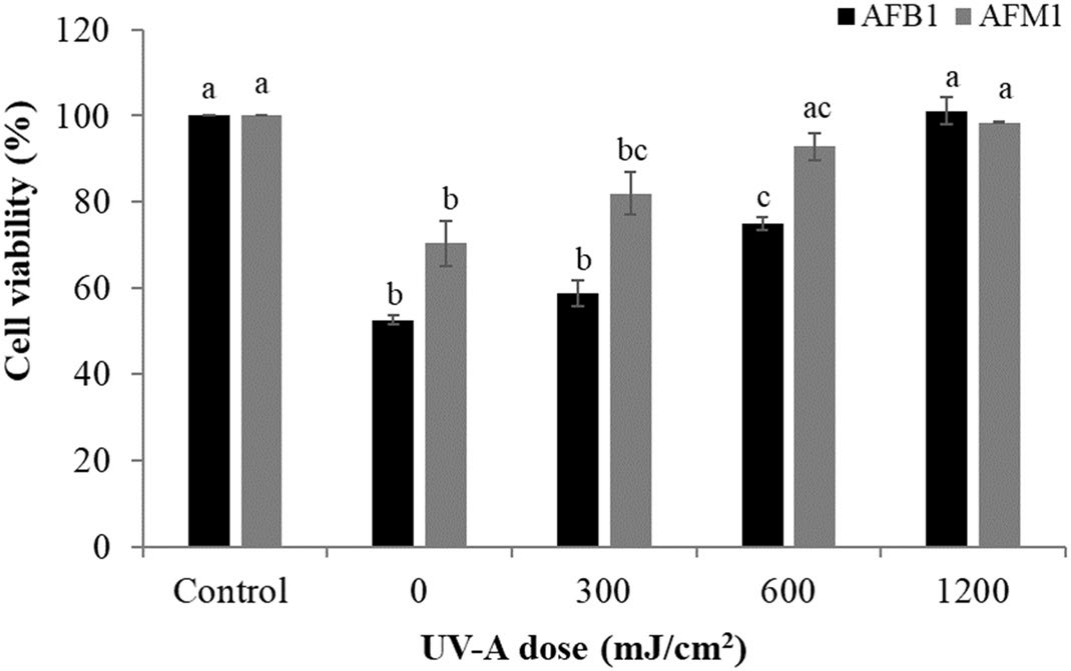
Cytotoxic effect of untreated and UV-A treated ultrapure water consists of (**a**) AFB_1_ (**b**) AFM_1_ on human hepatoma HepG2 cells. Results are expressed as mean percentage ± SD of two separate experiments. Levels connected by different letters are significantly different at *p* < 0.05.

The efficiency of the UV treatment in reducing the toxic potential of AFB_1_ and AFM_1_ was studied using in vitro cell culture methods. The cell viability was assessed using XTT [2,3-bis-(2methoxy-4-nitro-5-sulfophenyl)-2*H*-tetrazolium-5carboxanilide] assay, and the results were given in Fig. 5.

The cells were exposed to the test samples for 48 h. With an increase in the UV-A dose from 0 to 1,200 mJ/cm^2^, the cell viabilities increased from 52.5 ± 1.1 to 101.1 ± 3.2% in the samples treated with AFB1 respectively. Similarly, when the UV-A dose increased from 0 to 1,200 mJ/cm^2^, the cell viabilities increased from 70.42 ± 5.25 to 98.44 ± 0.25% in the samples treated with AFM_1_ respectively. The difference in cell viability between the treated and the control samples were found to be statistically significant. As the doses increased from 0 to 1,200 mJ/cm^2^, there is a significant decrease in the concentration of AFB_1_ (p value 0.001) and AFM_1_ (p value 0.0027), and thereby increase in cell viabilities was observed. Also, it is worth mentioning that there is no significant difference between the negative control and the maximum applied dose of 1,200 mJ/cm^2^ for both AFB_1_ (p value 0.76) and AFM_1_ (p value 0.899), clearly demonstrating the efficiency of UV-A degradation.

## Conclusion

The current research clearly demonstrated the efficiency of UV-A light in photolysis of AFB_1_ and AFM_1_ in ultrapure water. The results show that AFB_1_ and AFM_1_ were significantly reduced with increase in UV-A dose. A reduction of 70% and 84% were observed at 1,200 mJ cm^−2^ respectively. Likewise, cytotoxicity analysis of UV-A treated samples using HepG2 liver cells show increase in cell viability as the dose increases from 0 to 1,200 mJ/cm^2^ and no cytotoxicity was observed at UV-A dose of 1,200 mJ/cm^2^. These results confirm that the degraded AFB_1_ and AFM_1_ products of UV-A photolysis in water are safe without any effect on cell viability. Overall the results revealed that efficient degradation of AFB_1_ and AFM_1_ using UV-A irradiation at 365 nm could be a potential approach to reduce their levels in contaminated water. Due to its lipophilic nature, milk and edible vegetable oils are typically contaminated by aflatoxins hence could be reduced by UV-A light. Further studies will be conducted to assess the efficiency of this technology on reduction of AFB_1_ and AFM_1_ levels, quality (nutritional and sensory) and safety of the food products (milk and edible vegetable oils). Since AFB_1_ and AFM_1_ have absorbance maxima at 365 nm and milk and edible vegetable oils have absorbance minima at 365 nm, this permits efficient degradation of AFB_1_ and AFM_1_ by improving light penetration while potentially having less impact on the nutritional and sensory quality of food. UV-A dose response curves of aflatoxins will be generated using a continous flow UV system, continuous flow reactors are significantly more desirable for industrial food processes. 

## Supplementary information

Supplementary Information.
