# Supplementary material for: Performance of a UV-A LED system for degradation of aflatoxins B1 and M1 in pure water: kinetics and cytotoxicity study
Source: Sci Rep. 2020 Aug 10;10:13473. doi: 10.1038/s41598-020-70370-x (PMC7417570; doi:10.1038/s41598-020-70370-x)
Supplement: Supplementary file 1 — Supplementary Information. [file 41598_2020_70370_MOESM1_ESM.docx]

Performance of a UV-A LED system for degradation of Aflatoxins B_1_ and M_1_ in pure water_:_ kinetics and cytotoxicity study

Judy Stanley^a^, Ankit Patras^a*^, Brahmaiah Pendyala^a*^, Matthew J. Vergne^b^, Rishipal R Bansode^c^

^a^Food Biosciences and Technology Program, Department of Agricultural and Environmental Sciences, Tennessee State University, Nashville, 37209, TN, USA

^b^Department of Pharmaceutical Sciences and Department of Chemistry and Biochemistry, Nashville, 37204, TN, USA

^c^Center for Excellence in Post-Harvest Technologies, North Carolina Research Campus, North Carolina A&T State University, Kannapolis, NC, USA

**LC-MS SIM chromatographic peak areas of aflatoxins and degraded products**

(a)

(b)

(c)

(d)

(e)

(f)

**Figure S1.** The effect of UV dose on SIM chromatographic peak areas of aflatoxins and degraded products. (a) AFB_1_ (b) AFM_1_ (c) AFB_1_ - degraded product m/z 331 (d) AFB_1_ - degraded product m/z 303 (e) AFM_1_-degraded product m/z 347, RT 2.0 min (f) AFM_1_-degraded product m/z 347, RT 2.0 min
